# Metformin Alleviates Obesity and Systemic Oxidative Stress in Obese Young Swine

**DOI:** 10.3390/ph13070142

**Published:** 2020-07-06

**Authors:** Susana Astiz, Antonio Gonzalez-Bulnes, Isabel Astiz, Alicia Barbero, Jose Luis Pesantez-Pacheco, Consolacion Garcia-Contreras, Marta Vazquez-Gomez, Ana Heras-Molina

**Affiliations:** 1 INIA-Madrid, Avda. Puerta de Hierro s/n, 28040 Madrid, Spain; bulnes@inia.es (A.G.-B.); jose.pesantez@ucuenca.edu.ec (J.L.P.-P.); garcia.consolacion@inia.es (C.G.-C.); delasheras.ana@inia.es (A.H.-M.); 2 Facultad de Veterinaria, Universidad Complutense de Madrid, Ciudad Universitaria s/n, 28040 Madrid, Spain; mvgomez@ucm.es; 3 Unidad de Pediatría, Atención Primaria, Centro de Salud Puerta Bonita, Calle de la Alegría 24, 28025 Madrid, Spain; astiz.isabel@gmail.com; 4 Servicio de Diagnostico por Imagen, Universidad Alfonso X El Sabio, Avenida de la Universidad 1, 28691 Villanueva de la Cañada, Spain; aliciabarbero.vet@gmail.com; 5 Escuela de Medicina Veterinaria y Zootecnia, Facultad de Ciencias Agropecuarias, Universidad de Cuenca, Avda. Doce de Octubre, 010220 Cuenca, Ecuador

**Keywords:** metformin, obesity, oxidative-stress, pediatrics

## Abstract

The present study assessed the relationship between obesity induced by lifestyle and systemic oxidative stress and possible modulations by oral metformin treatments in young individuals, by using a translational swine model of obesity and associated cardiometabolic disorders (Iberian pig). The results indicate the existence of an age-related increase in both adiposity and systemic oxidative stress (using hydrogen peroxide as a marker), which is higher in individuals with obesogenic lifestyle and increased weight and obesity. Such effect was not found in individuals treated with metformin. The translation of these results suggests that childhood obesity increases production of reactive oxygen species (ROS), and therefore systemic oxidative stress. Treatment with metformin would improve such oxidative status.

## 1. Introduction

Childhood obesity has been traditionally associated with affluent social environments in highly developed countries. Currently, it is scattering with respect to children from all social statuses in both developed and developing countries. Hence, there is an urgent necessity to tackle this health problem; mainly because overweight and obese children are prone to develop cardiometabolic disorders in infancy, youth and adulthood [1].

The pathogenic mechanisms of the metabolic alterations associated with obesity seem to be mainly caused by insulin resistance and oxidative stress [2], since obesity clearly increases systemic oxidative stress, through increased production of reactive oxygen species (ROS). Specifically, in children, a recognized marker of systemic oxidative stress like the urine hydrogen peroxide is augmented with increases in weight, body mass index and waist circumference [3].

Nowadays, pharmacological treatments for avoiding obesity and associated diseases in children are based on the use of oral anti-hyperglycemic drugs; mainly metformin [3-(diaminomethylidene)-1,1-dimethylguanidine], combined with adequate nutrition and exercise [1]. Metformin improves insulin sensitivity, but there is also evidence of modulatory effects on oxidative stress in adult rodents [4]. There are clear evidences of positive effects on oxidative stress damage by decreasing reactive oxygen species (ROS) production via AMPK activation and through inhibition of nicotinamide adenine dinucleotide phosphate [NAD(P)H] oxidase and the respiratory mitochondrial chain [5,6]. However, there is a lack of information on its possible usefulness in mammalian species other than rodents, including humans, and in younger individuals.

Thus, we aimed to determine the relationship, in young individuals, between obesity induced by lifestyle and systemic oxidative stress and possible effects of oral metformin treatments. Systemic oxidative stress was assessed using plasma concentrations of hydrogen peroxide as a marker. Hydrogen peroxide is a hazardous reactive oxygen species (ROS) against tissues and cells, which blood concentrations are augmented with in case of obesity [2].

Most of the data obtained on pathophysiology and treatment of childhood obesity have been acquired by observational studies, which are usually biased by concurrent intrinsic ethnic features [7,8] and socio-cultural, economic and behavioral factors [9,10,11]. Hence, there is a necessity of data from interventional research; however, interventional experimentation in humans is limited by ethical issues and therefore it needs to be performed in animal models. The pig is an outstanding model for translational studies in obesity and associated disorders. Pigs share more anatomical (including proportional organ sizes) and physiological similarities (including lifestyle: diurnal rhythms, omnivorous habits and propensity to sedentary behavior) with humans than any other animal species, except for primates [12,13].

Hence, the pig has been considered a unique animal model for studies linking nutrition, metabolism, reproduction and development a since long time ago [14]. There are even different swine breeds and strains for different biomedical research objectives [13]. Specifically, a group of swine breeds, known as fatty-pigs (with the most representative ones being the Iberian and Mangalica breeds), are currently characterized as a robust, amenable and reliable translational model for conducting studies on obesity, metabolic syndrome and other nutrition-associated diseases in humans [15,16]. In fact, the Iberian pig is characterized as an outstanding model for translational metabolic studies in pediatrics, showing juvenile adiposity and precocious puberty [15,17,18]. The use of metformin treatments during juvenile development of Iberian gilts has positive effects on growth patterns, muscle deposition, adiposity and metabolic features [19].

## 2. Results

### 2.1. Changes in Growth Patterns and Adiposity

**Figure 1:**
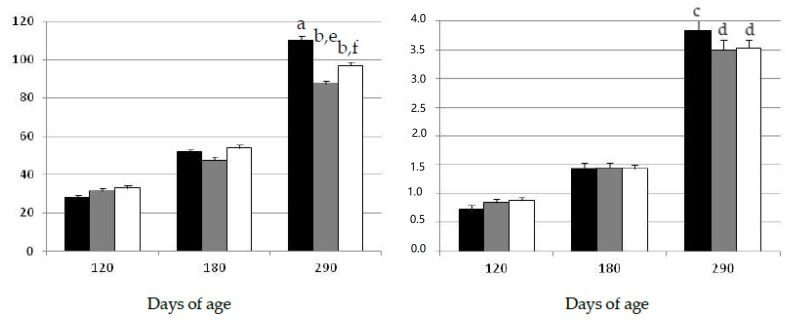
Changes over time in the mean values (±S.E.M.) for body mass (left hand) and subcutaneous fat depth (right hand) in gilts confined and fed ad libitum with obesogenic diets (group OB, black bar) and gilts with opportunity of exercise and controlled non-obesogenic diets without or with metformin (groups EXE and MET, grey and white bars, respectively). Significant differences are denoted by different superscript letters among treatments (a ≠ b: *p* < 0.001; c ≠ d: *p* < 0.005; e ≠ f: *p* < 0.05).

The assessment of the data showed that both body weight and subcutaneous back-fat depth increased during the experimental period, throughout the juvenile period until early-adulthood at 290 days-old (Figure 1). There were no significant differences among groups in body corpulence and adiposity at the start of the study at 120 days of age. Afterwards, the assessment of body mass and adiposity two months later (at 180 days of age) again showed no significant differences among groups during the period corresponding to juvenile development, in spite of the different diet and lifestyle.

Changes in growth and adiposity were observed later, at adulthood (290 days of age), when the sows in the group exposed to the obesogenic diet (group OB) showed a significantly higher body-mass (*p* < 0.001) and more subcutaneous fat (*p* < 0.005) than the sows in the groups under the maintenance diet and treated with metformin (groups EXE and MET, respectively). Moreover, the gilts in the group MET were heavier than the gilts in the group EXE (*p* < 0.05), although the amount of subcutaneous fat was similar in both groups.

Similarly, the visceral fat depots were greater in the group OB than in the groups EXE and MET (*p* < 0.05 for both), without significant differences between these two last groups (Table 1).

### 2.2. Changes in Oxidative Status

**Figure 2:**
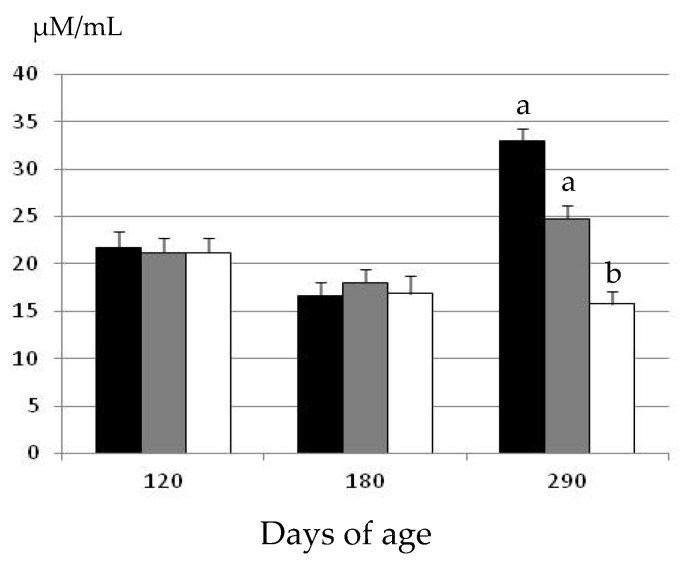
Changes over time in the mean values (± S.E.M.) for plasma hydrogen peroxide concentrations in gilts confined and fed ad libitum with obesogenic diets (group OB, black bar) and gilts with opportunity of exercise and controlled non-obesogenic diets without or with metformin (groups EXE and MET, grey and white bars, respectively). Significant differences are denoted by different superscript letters among treatments (a ≠ b: *p* < 0.05).

The assessment of plasma hydrogen peroxide concentrations at the start of the experimental period showed similar concentrations among the three groups at 120 days of age (Figure 2).

There were again no significant differences in plasma hydrogen peroxide concentrations at 180 days of age. Conversely, there were significant differences among groups at 290 days of age. There were no age-related changes in the gilts treated with metformin (group MET), and plasma hydrogen peroxide concentrations remained similar at 180 and 290 days of age. Conversely, plasma hydrogen peroxide concentrations increased with age in the gilts belonging to the groups EXE and OB (*p* < 0.05 and *p* < 0.01, respectively). Hence, at 290 days of age, the values in the group MET were significantly lower than in the groups EXE and OB (*p* < 0.05). Moreover, the concentrations of plasma hydrogen peroxide were also higher, around 25%, in the group OB than in the group EXE; however, the difference did not reach statistical significance.

## 3. Discussion

The present study indicates that the systemic oxidative stress in a swine model with genetic predisposition to obesity increases with the increment in adiposity occurring during the late-juvenile development. There were no significant differences in plasma concentrations of hydrogen peroxide when comparing early-juvenile age-stages (120 and 180 days of age), independently of the group of treatment. However, there were significant differences in oxidative stress when comparing 180 and 290 days of age concomitantly with a significant increase in obesity, as indicated by differences in both the subcutaneous and visceral fat depots (from 1.4 cm to above 3.5 cm and from 17 to 31 cm^2^, respectively).

Such increase in oxidative stress was exacerbated in animals with higher weight and adiposity due to inadequate lifestyle (obesogenic diet and lack of exercise). Although further studies, with a higher number of animals and sampling, are necessary to determine the causal mechanisms of this finding, our results contribute to support previous evidences addressing that childhood obesity increases production of ROS and, therefore, systemic oxidative stress [3].

The emerging evidence pointing out that increased oxidative stress is a direct consequence of adiposity and that increased oxidative stress and insulin resistance are early instigators of metabolic syndrome indicate the necessity of therapeutic strategies focused on diminishing both disorders. In human pediatrics, it is well-known that insulin resistance decreases with adequate exercise and diet [20,21]. However, clinical practice includes the use of metformin [5,22], mainly due to the development of more acute disorders in children than in adults [1], and the lack of adherence to lifestyle regulations when compared to pharmacological treatments, which favours results with metformin [23,24,25,26,27,28,29].

The results of the current study indicate that metformin therapy in a swine model of obesity, besides modulating metabolic features as previously reported [19], would also improve the oxidative status during the late stages of juvenile development.

Our results reinforce the data found in recent studies in adult rodents [4] and, in combination with previous results in the same model [19], suggest that metformin, avoiding fat deposition and favouring muscle development, would favour the general homeostasis and the health status of the individual. The administration of metformin in the present experiment showed to have a direct effect on the performance of the gilts. The sows in the group with opportunity of exercise and treated with metformin were heavier than the sows in the group with only opportunity of exercise and no metformin treatment. However, adiposity was similar in both groups, suggesting that metformin treatment enhanced body development mainly through increased muscle deposition.

The intrinsic mechanism cannot be elucidated under the experimental conditions of the current study. There are no specific studies about possible effects of metformin on muscle development in growing individuals. However, previous data on the effects of metformin on muscle development indicate that the compound attenuates the loss of muscular mass in patients with severe burn injuries or sarcopenia [30,31]. The mechanism of this effect is not yet known but may be related to increases in net muscle protein anabolism by enhancing the availability of energy within the muscle.

Metformin lowers plasma glucose mainly by decreasing hepatic glucose production; mainly as a result of a reduced gluconeogenesis [32,33]. However, a concomitant mechanism of metformin for lowering plasma glucose is the improvement of insulin sensitivity (i.e., favoring uptake of glucose by the muscle and other tissues [33]). In this scenario, an increased uptake of glucose by the muscles would favor its development by increasing its available energy by such higher peripheral glucose disposal [34]. Moreover, metformin improves insulin sensitivity and glucose uptake through activation of AMP-activated protein kinase (AMPK; [35]), a cellular energy sensor activated under metabolic stress [36,37] and by enhanced gene expression of peroxisome proliferator-activated receptors [38], which are involved in myogenesis and muscle development [39,40]. Moreover, the major downstream target of AMPK is the mammalian target of rapamycin (mTOR), a kinase whose activity is important in cellular growth processes and protein synthesis [41,42]. Hence, metformin would also directly improve muscle development through the enhancement of tissue growth.

## 4. Material and Methods

### 4.1. Ethics Statement

The present experiment, involving Iberian gilts, was carried out at the INIA animal facilities, according to the EU Directive about the protection of animals used for scientific purposes (2010/63/UE). These facilities meet the local, national and European requirements for Scientific Procedure Establishments.

The experimental procedures were assessed and approved by the INIA Committee of Ethics in Animal Research (report CEEA2010/003). Animals were managed according to the Spanish Policy for Animal Protection (RD 53/2013), which meets the European Union Directive 2010/63/UE on the protection of research animals.

### 4.2. Animals and Experimental Design

The study involved 24 Iberian gilts, housed in a collective pen with 1 m^2^ of surface per animal, which were fed ad libitum with a high-fat diet (6.3% of fat content and 3.36 Mcal/kg of metabolizable energy) from 60 to 120 days-old for allowing the development of obesity. Food consumption was estimated to be around 1.5–2 kg/day per animal, depending on age. At 120 days-old, these gilts were randomly distributed in three equal experimental groups. A first group (group OB, *n* = 8) was maintained in a pen with restricted space (3 m^2^ of surface per animal, which is still in agreement with animal welfare regulations) and continued being fed ad libitum with the high-fat diet. Food consumption in this group was estimated to be 2–4.5 kg/day per animal, depending on age. A second group (group EXE, *n* = 8) was maintained in a pen with 7 m^2^ of surface per animal (between seven-fold and 12-fold, depending on age, of the surface indicated by welfare regulations), allowing exercise, and was fed with a standard diet (2.8% of fat and 3.08 Mcal/kg of metabolizable energy) for fulfilling maintenance requirements. The amount of food offered was recalculated according to age to fulfill daily maintenance requirements (from 1.5–2.5 kg/day per animal). A third group (group MET, *n* = 8) was maintained in similar pens and fed with similar diets to group EXE, but received a daily dose of metformin (850 mg; Dianben^®^, Merck Serono, Madrid, Spain), by individual top-dressing over the morning feed.

### 4.3. Evaluation of Growth Patterns, Adiposity and Oxidative Status

**Figure 3:**
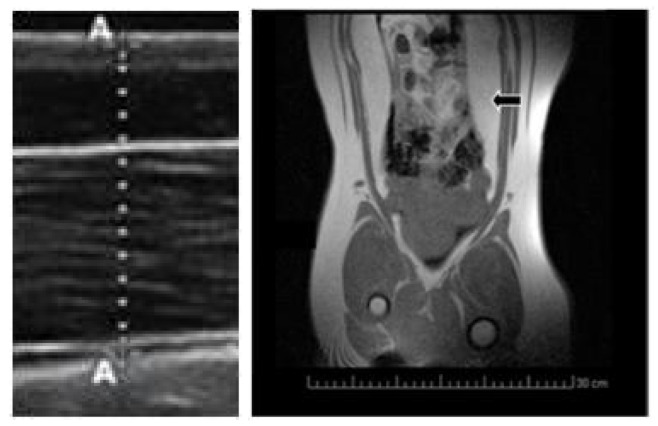
Imaging of subcutaneous fat depth by ultrasonography (left hand) and visceral fat depots by magnetic resonance imaging (MRI; right hand; black arrow).

Body mass was measured at three time-points during the juvenile development (120, 180 and 290 days of age). Subcutaneous back-fat depth was evaluated by ultrasonography (Figure 3) with a SonoSite S-Series ultrasound machine equipped with a 5–8 MHz linear array probe (SonoSite Inc., Bothell, WA, USA), as previously described [43]. Concomitantly, a blood sample was drawn by jugular venopuncture with 5 mL sterile heparin blood vacuum tubes (Vacutainer^TM^ Systems Europe; Becton Dickinson, Meylan Cedex, France) and centrifuged at 1500 g for 15 min in order to obtain the plasma. Plasma concentrations of hydrogen peroxide were determined with an enzyme immunoassay kit (Abcam plc, Cambridge, UK), with a detection limit of 30 nm/mL. Finally, visceral fat depots were assessed at 290 days old by magnetic resonance imaging (Figure 3) with a Panorama 0.23 T scanner with a body/spine XL coil (Philips Medical Systems, Best, the Netherlands) as previously described [19].

### 4.4. Statistical Analyses

The effects of treatments on body mass, fat content and oxidative status were assessed by analysis of variance for repeated measures (split-plot ANOVA), whilst possible differences in the values of visceral fat depots were assessed by ANOVA. Results were expressed as the means ±S.E.M., and statistical significance was accepted from *p* < 0.05.

## 5. Conclusions

The results of the present study suggest that higher weight and adiposity due to inadequate lifestyle (obesogenic diet and lack of exercise) during childhood and late juvenile development increase systemic oxidative stress. There are positive effects of lifestyle (diet and exercise) on obesity traits which, if combined with the administration of metformin would also modify the oxidative status of the individual, diminishing the production of ROS and, therefore, the systemic oxidative stress.

## Figures and Tables

**Table 1:** Mean values (± S.E.M.) for visceral fat depots in gilts with opportunity of exercise and controlled non-obesogenic diets, treated or not with metformin (groups EXE and MET, respectively) and gilts confined and fed ad libitum with obesogenic diets (group OB).

	Group OB	Group EXE	Group MET
**Visceral Fat Depot (cm^2^)**	31.2 ± 5.3^a^	18.3 ± 3.4^b^	17.2 ± 4.1^b^

Significant differences are denoted by different superscript letters among treatments (a ≠ b: *p* < 0.05).
